# Telangiectasia Macularis Eruptiva Perstans in a 55-Year-Old Female With Type 2 Diabetes Mellitus

**DOI:** 10.7759/cureus.57333

**Published:** 2024-03-31

**Authors:** Norah S Alhammad, Sumayah Alshehri, Toleen M Alawadi, Ali Ragaban, Faisal S Alyahya, Ahmad Alharbi

**Affiliations:** 1 Department of Dermatology, King Fahad Armed Forces Hospital, Jeddah, SAU; 2 Department of Medicine, King Abdulaziz University, Jeddah, SAU; 3 Department of Dermatology, Taibah University, Madinah, SAU

**Keywords:** telangiectasia macularis eruptiva, clinical dermatology, rash, maculopapular cutaneous mastocytosis, cutaneous mastocytosis, mastocytosis, tmep

## Abstract

The authors would like to report a rare case of telangiectasia macularis eruptiva perstans (TMEP), a form of cutaneous mastocytosis, in a 55-year-old female patient with a recent diagnosis of type 2 diabetes mellitus on empagliflozin. The patient presented with a two-month history of rash and itching on her lower extremities, unresponsive to topical treatment. A dermoscopic evaluation and a skin biopsy confirmed the diagnosis of TMEP. The patient demonstrated significant improvement with antihistamine and topical steroid treatment.

## Introduction

Telangiectasia macularis eruptiva perstans (TMEP) is a rare form of cutaneous mastocytosis characterized by persistent telangiectatic macules and patches [1,2]. From bone marrow progenitor cells, mastocytes migrate to tissues, where they play a role in cellular immunity, inflammation, and the metabolism of connective and osseous tissues [3]. Mastocytosis develops as a result of their proliferation [3]. Recognizing TMEP can be challenging for dermatologists and is often overlooked [1]. Its atypical clinical presentation can sometimes be mistaken for an allergic reaction [1]. A conclusive diagnosis usually requires a skin biopsy, while dermoscopic analysis may provide insights [1]. Giemsa and toluidine blue are two examples of special stains that are used to highlight the presence of mast cells by showing their metachromatic cytoplasmic granules [4]. This case report emphasizes the significance of taking TMEP into account when making a differential diagnosis of patients with persistent rashes of unknown etiology.

## Case presentation

A 55-year-old female with a three-month history of type 2 diabetes mellitus (DM), managed with empagliflozin 10 mg once daily, presented with a two-month history of rash and itching on the lower extremities. The itching was worse at night, interfering with sleep, and not responsive to topical treatments. There was no involvement of other body areas, no previous history of similar complaints, no contact history with ill patients, no family history of similar complaints, and no history of recent medication use except for empagliflozin. The patient denied any constitutional symptoms such as fever, weight loss, fatigue, or night sweats. There was no history of nausea, vomiting, or abdominal pain. The systematic review was otherwise unremarkable.

**Figure 1:**
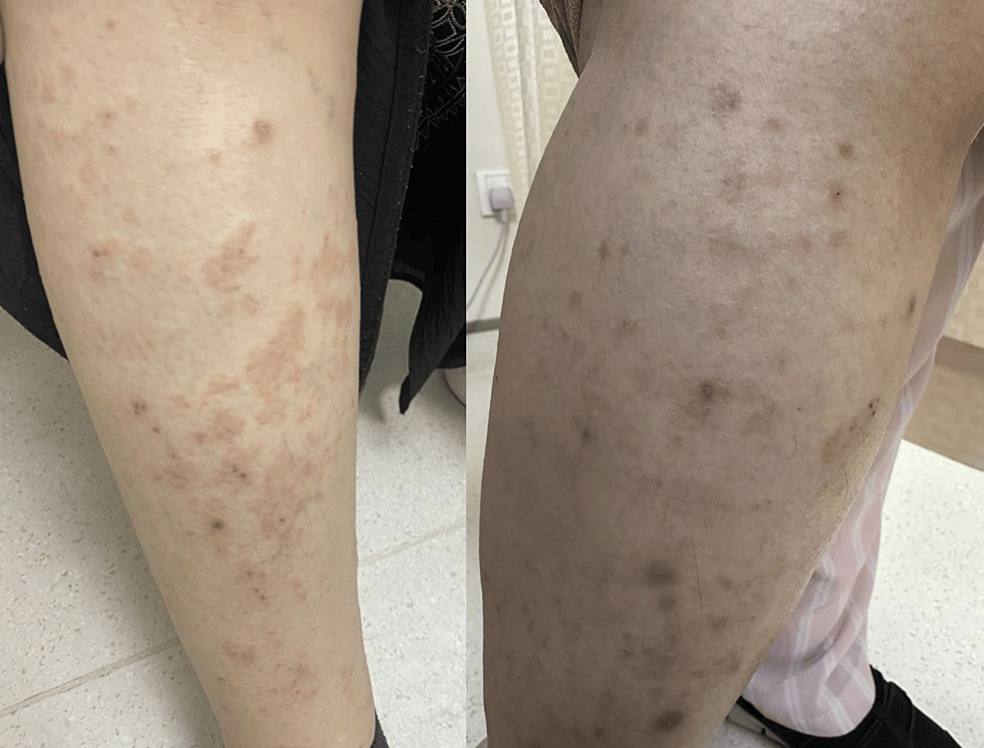
Ill-defined orange to tan-brown telangiectatic patches and macules with some excoriated papules

On physical examination, the patient was alert, conscious, and vitally stable. The anterior shaft of the lower extremities revealed ill-defined orange to tan-brown telangiectatic patches and macules, with some excoriated papules (Figure 1). Darier's sign was negative; no other abnormalities were noted in other body areas, such as lymph nodes, scalp, nails, or mucous membranes. A dermoscopic examination showed a reticular vascular pattern.

**Figure 2:**
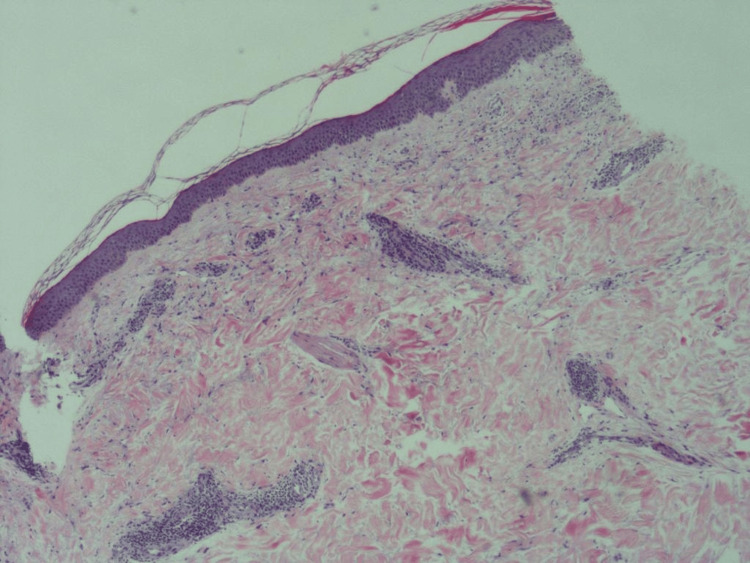
Perivascular and superficial lymphocytic infiltrates with heavy densities of mast cells, seen by 4x magnification

**Figure 3:**
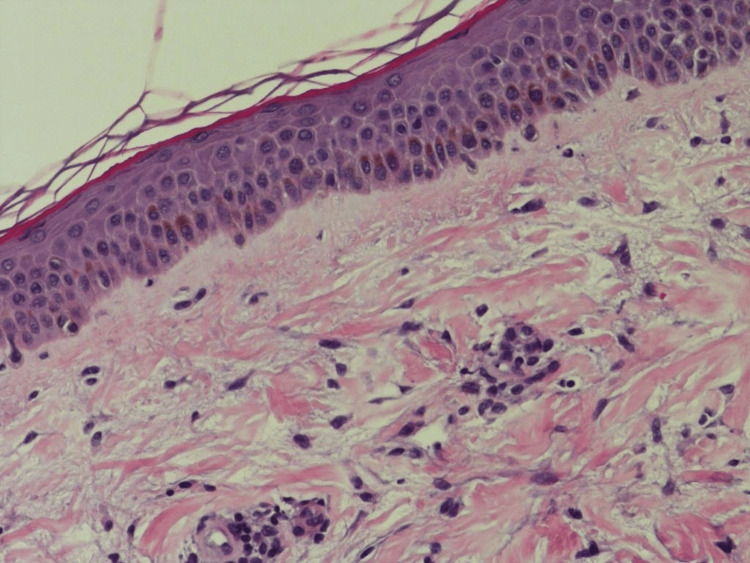
Basal hyperpigmentation, seen by 20x magnification

**Figure 4:**
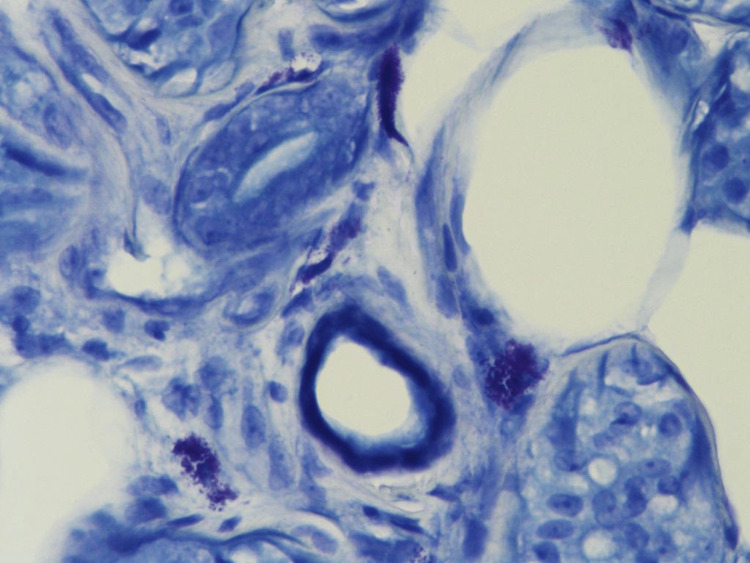
Giemsa stain highlights mast cell predominance

**Figure 5:**
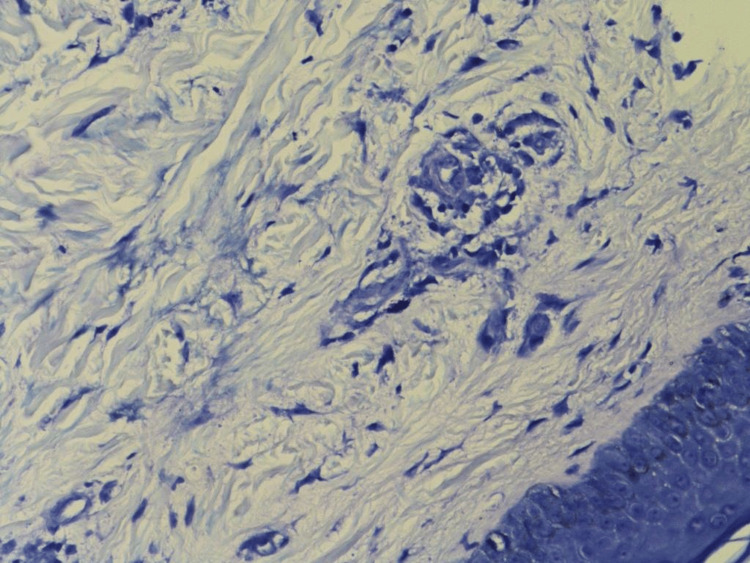
No fungal elements using PAS PAS: periodic acid-Schiff

A 3 mm skin punch biopsy was taken from the left leg under local anesthesia and demonstrated perivascular lymphocytic infiltration with heavy densities of mast cells and basal hyperpigmentation, suggestive of TMEP (Figures 2-3). Special stains periodic acid-Schiff (PAS) and Giemsa were done with controlled measures showing appropriate reactivity. Giemsa stain highlights the predominance of mast cells (Figure 4). PAS was negative for fungal elements (Figure 5). The patient was educated on avoiding triggers and possible trigger factors. She was prescribed desloratadine (5 mg) once daily at night and a topical steroid. At the follow-up visit, the patient reported significant improvement in itching and rash, with near resolution of the lesions.

## Discussion

This case report describes a rare presentation of TMEP in a patient with type 2 DM. In our patient's case, Darier's sign was negative. However, in another case report that was published in 2014, they mentioned that the Darier sign was found positive, which is considered to be a rare presentation of TMEP, and it shouldn’t guide you into excluding TMEP [5]. There was no systemic involvement in our patient. Despite what was mentioned in another study done in Turkey, they presented that it is common to have systemic involvement in older patients [5]. The diagnosis of TMEP was confirmed via dermoscopy and a 3 mm skin punch biopsy.

The patient showed a marked response to antihistamine and topical steroid treatment. Other literature introduced new regimens for treating TMEP flares. An article published in 2021 suggested the use of omalizumab as a potential treatment for chronic recurrent flares of TMEP [6]. Another study done in 2017 showed an improvement in both functional and cosmetic aspects after the use of psoralen and ultraviolet A therapy, total skin electron beam radiation, and flashlamp pulsed-dye laser treatment [7].

We would like to suggest a hypothesis regarding the association of TMEP flares after taking empagliflozin, which is used for the management of DM since our patient started experiencing the symptomatic flares almost three months after starting empagliflozin, and this may be a potential trigger for this case.

Two distinct articles reported the emergence of a skin rash that was not specified by biopsy, which started after prescribing sodium-glucose cotransporter-2 inhibitors for a diabetic patient, and the rash resolved after stopping the medication; the association was yet to be cleared between the medication and rash [8,9].

It is essential for clinicians to be aware of TMEP as a potential diagnosis when encountering patients with persistent rashes of unknown etiology. Early recognition and management can improve patient outcomes and alleviate symptoms.

## Conclusions

TMEP is a rare form of cutaneous mastocytosis that should be considered in patients presenting with persistent and unexplained rashes. A dermoscopic examination and skin punch biopsy can aid in the diagnostic process of TMEP. Antihistamine and topical steroid treatments can provide symptomatic relief and improve patient outcomes, and we recommend applying more studies on the potential triggers of TMEP, such as introducing a new medication, and the possible treatment options other than the use of antihistamines and topical steroids.
